# Stereotactic cysto-ventricular catheters in craniopharyngiomas: an effective minimally invasive method to improve visual impairment and achieve long-term cyst volume reduction

**DOI:** 10.1007/s10143-021-01510-8

**Published:** 2021-03-05

**Authors:** Christine Steiert, Juergen Grauvogel, Roland Roelz, Theo Demerath, Daniel Schnell, Juergen Beck, Volker Arnd Coenen, Peter Christoph Reinacher

**Affiliations:** 1 Department of Neurosurgery, Medical Center - University of Freiburg, Faculty of Medicine, University of Freiburg, Freiburg, Germany; 2 Department of Neuroradiology, Medical Center - University of Freiburg, Faculty of Medicine, University of Freiburg, Freiburg, Germany; 3 Department of Radiation Oncology, Medical Center - University of Freiburg, Faculty of Medicine, University of Freiburg, Freiburg, Germany; 4 Department of Stereotactic and Functional Neurosurgery, Medical Center - University of Freiburg, Faculty of Medicine, University of Freiburg, Breisacher Str. 64, D-79106 Freiburg, Germany; 5 Fraunhofer Institute for Laser Technology, Aachen, Germany

**Keywords:** Craniopharyngioma cyst, Minimally invasive, Stereotactic catheter, Ventriculo-cystostomy, Cysto-ventricular shunting

## Abstract

Craniopharyngiomas are typically located in the sellar region and frequently contain space-occupying cysts. They usually cause visual impairment and endocrine disorders. Due to the high potential morbidity associated with radical resection, several less invasive surgical approaches have been developed. This study investigated stereotactic-guided implantation of cysto-ventricular catheters (CVC) as a new method to reduce and control cystic components. Twelve patients with cystic craniopharyngiomas were treated with CVC in our hospital between 04/2013 and 05/2017. The clinical and radiological data were retrospectively analysed to evaluate safety aspects as well as ophthalmological and endocrine symptoms. The long-term development of tumour and cyst volumes was assessed by volumetry. The median age of our patients was 69.0 years and the median follow-up period was 41.0 months. Volumetric analyses demonstrated a mean reduction of cyst volume of 64.2% after CVC implantation. At last follow-up assessment, there was a mean reduction of cyst volume of 92.0% and total tumour volume of 85.8% after completion of radiotherapy. Visual acuity improved in 90% of affected patients, and visual field defects improved in 70% of affected patients. No patient showed ophthalmological deterioration after surgery, and endocrine disorders remained stable. Stereotactic implantation of CVC proved to be a safe minimally invasive method for the long-term reduction of cystic components with improved ophthalmological symptoms. The consequential decrease of total tumour volumes optimised conditions for adjuvant radiotherapy. Given the low surgical morbidity and the effective drainage of tumour cysts, this technique should be considered for the treatment of selected cystic craniopharyngiomas.

## Introduction

Craniopharyngiomas, as rare benign extra-axial tumours, account for approximately 1–3% of all intracranial neoplasms and are frequently associated with cystic formations [6, 23]. There are two peaks in age distribution, one occurring in childhood and the other in adults between 50 and 75 years of age [23]. Due to their location in the (para-)sellar region, these tumours often lead to significant clinical impairment by compression of the optic pathways, 3^rd^ ventricle, hypothalamus or pituitary stalk [11, 44].

Therapeutic management of craniopharyngiomas remains controversial and challenging. Complete tumour removal without neurological deterioration is difficult because of its proximity and adherence to adjacent eloquent neural structures [11, 44]. Despite the advances in modern microsurgery, radical resection is still associated with high potential morbidity [27, 29]. Consequently, more recent therapeutic strategies aim to reduce intracranial pressure and local tumour control with improvement or preservation of visual impairment as well as the pituitary gland and hypothalamic function [14, 23, 24, 26]. Therefore, several less invasive surgical strategies to treat space-occupying cystic components have been developed, usually followed by radiotherapy [28, 36, 39].

Besides endoscopic approaches with their respective limitations due to cyst location and recurrence [12, 18, 42], appropriate minimally invasive options are stereotactic procedures like cyst punctures or catheter placement with subcutaneous Ommaya reservoirs allowing repeated cyst aspirations [39]. In these procedures, frequent cyst recurrence is a common problem but long-term reduction of cyst volume by connection to the surrounding subarachnoid space or ventricular system has been reported [9, 17, 21, 34]. The present study investigated the specific effect of stereotactic transventricular implantation of cyst catheters establishing communication between the cyst cavity and ventricular system as a minimally invasive technique to reduce and control associated cystic components in craniopharyngioma patients.

## Materials and methods

### Patients

Patients selected for implantation of a CVC to control cyst size fulfilled the following criteria: suprasellar tumour (histologically confirmed or radiologically defined as craniopharyngioma) with accompanying space-occupying cystic formation, need for treatment because of the mass effect and/or ophthalmologic problems, no contraindication for surgery under general anaesthesia as well as normal haemostasis and no signs of infection, informed consent of patients or a legal guardian.

Patients were treated in the Department of Functional and Stereotactic Neurosurgery in a tertiary referral centre. The retrospective analysis was approved by the independent ethics committee of our medical centre (reference no. 432/20) and is reported according to institutional guidelines.

### Surgical procedure

**Fig. 1:**
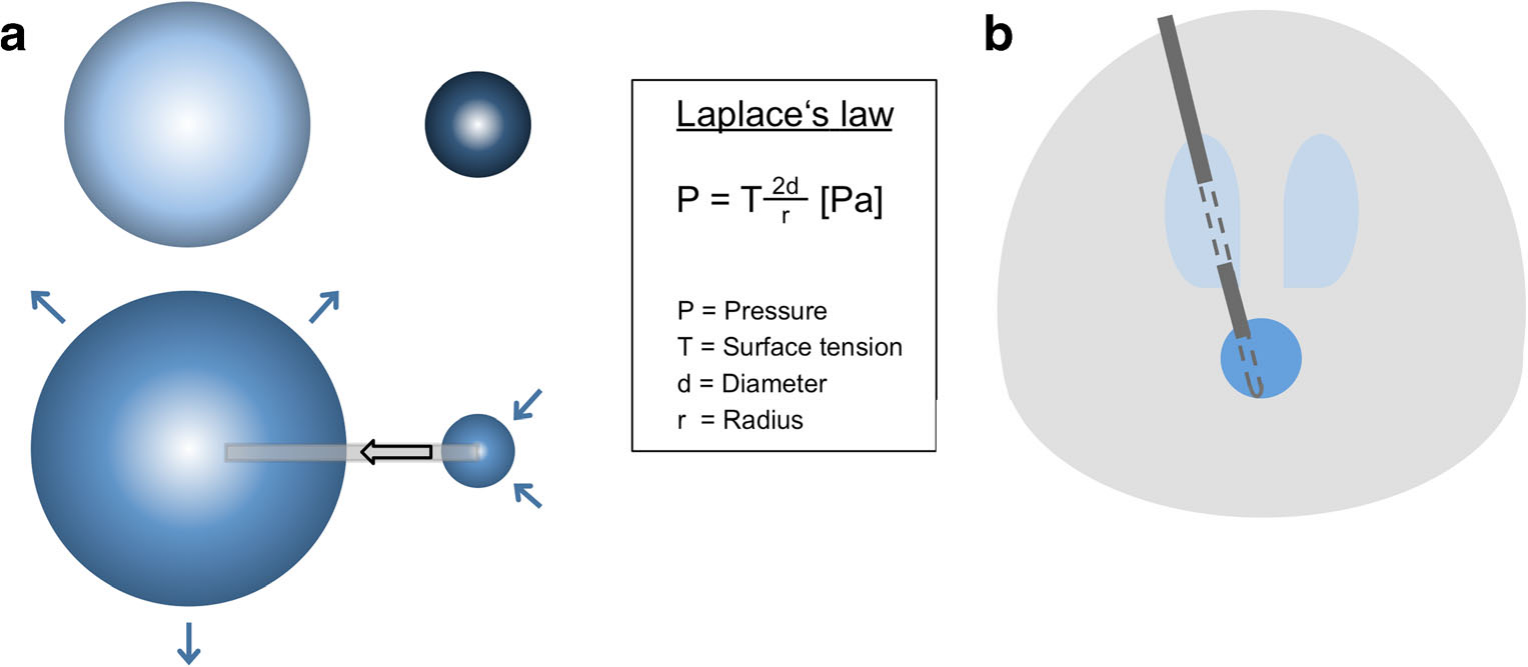
Laplace’s law and catheter scheme. Following Laplace’s law, after connection of a small sphere (dark blue) to a large sphere (light blue), a volume shift into the large sphere can be observed due to the different surface tensions (**a**). Scheme of a catheter connecting the small volume of craniopharyngioma cyst to the large volume of the CSF system via holes within the cyst and the lateral ventricle (**b**)

**Fig. 2:**
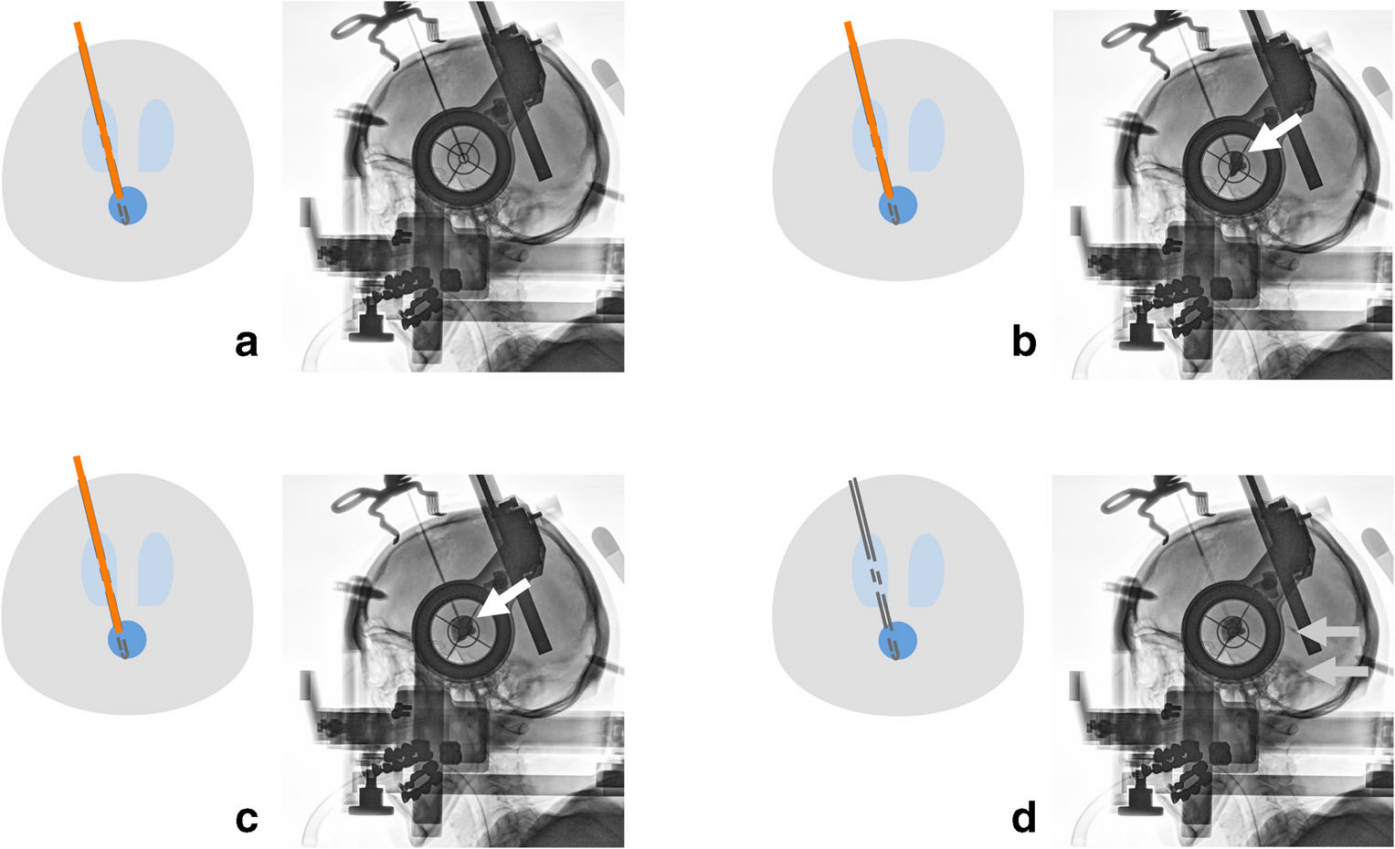
Stereotactic cysto-ventriculostomy and intraoperative ventriculography. Intraoperative lateral x-ray images demonstrate stereotactic transventricular catheter implantation (**a**), aspiration of cyst fluid and instillation of contrast agent into the cyst cavity (white arrow) (**b**), the whole cavity filled with contrast agent (white arrow), still without connection to the CSF system due to the inserted mandrin (**c**), and after mandrin removal, the contrast agent spreading with CSF from the cyst cavity to the ventricular system (light grey arrows) via the holes in the catheter connecting the cavity to the ventricle (**d**)

Patients were operated under general anaesthesia. All surgeries were performed with frame-based stereotactic guidance using a Leksell G-Frame (Elekta, Stockholm, Sweden). Preoperative contrast-enhanced 3D magnetic resonance images (MRI) and intraoperative computed tomography angiography images (performed with the stereotactic frame attached to the patients’ head) were transferred to the surgical planning station (Leksell SurgiPlan®) (Elekta, Stockholm, Sweden). Surgical trajectories to the cystic components were planned transventricularly (lateral ventricles). Standard burr holes were performed under stereotactic guidance. Afterwards, a standard ventricular catheter (typically 2.8 mm diameter) stabilised by an internally located stereotactic cannula and an additional solid steel mandrin was inserted for transventricular cyst puncture under stereotactic guidance with the endpoint of the tip lying in the cyst cavity. Following Laplace’s law of fluid mechanics, permanent spontaneous drainage of cysts should be achieved via connection to the ventricular system (see Fig. 1). An x-ray control showed the catheter tip located within the endpoint. First, the mandrin was removed, and after slow aspiration of 1 ml of cyst fluid, 1 ml of contrast agent (iodine-based x-ray contrast agent Solutrast® 200 M) was carefully injected within the cavity. An x-ray control showed the cyst filled with contrast-enhanced fluid. Then, the stereotactic cannula was removed to enable free communication of the cyst cavity with the ventricular system. Another x-ray control demonstrated the distribution of the contrast agent within the ventricular system. Afterwards, the distal end of the catheter was closed with a titanium plug (Aesculap, Tuttlingen, Germany) and fixed to the skull with a titanium microfixation system (Aesculap), using one microplate (12 mm length) and two microscrews (1.5 mm diameter and 4 mm length) to prevent dislocation. Figure 2 shows the surgical steps with the corresponding intraoperative x-ray images.

In patients where a concurrent biopsy for histological confirmation was performed during CVC implantation, samples were first taken from the solid tumour using a Sedan 2.1-mm outer diameter side-cutting biopsy needle (Elekta, Stockholm, Sweden). Biopsies and CVC implantation were performed via the same trajectory, if possible, alternatively, a further non-transventricular trajectory was planned to reach the solid tumour before CVC implantation.

### Outcome parameters

**Fig. 3:**
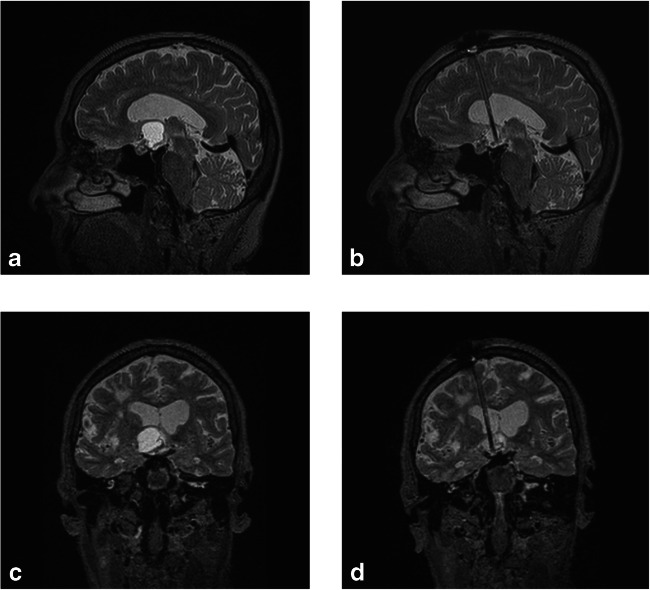
Preoperative sagittal (**a**) and coronal (**c**) T2-weighted MR images compared to postoperative sagittal (**b**) and coronal (**d**) T2-weighted MR images. Preoperative images (**a**, **c**) demonstrate the T2 hyperintense protein-rich craniopharyngioma cyst bulging above into the ventricular system. Postoperative images (**b**, **d**) demonstrate the volume reduction of the CSF-isointense cyst connected to the ventricular system via the implanted catheter

Three-dimensional MR imaging (3D MRI) for preoperative volumetric measurement and surgical planning was performed within 1 week before surgery. Routine postoperative MRI, performed between days 1 and 3 after surgery, documented the catheter position as well as the cyst size for postoperative volumetric analysis. Figure 3 shows the pre- and postoperative MR images with effective cyst drainage by the inserted catheter. Follow-up measurements of cyst and tumour volumes were performed using the last available follow-up MRI. Volumetric analyses based on 3D MRI were manually performed using the open-source software OsiriX (http://www.osirix-viewer.com), with average values calculated based on the measurement of each volume by two independent physicians.

Following our standard procedure, the ophthalmological status was assessed pre- and postoperatively within 1 week prior to and after surgery, respectively. To detect potential postoperative diabetes insipidus, there was frequent monitoring of fluid balance, urine specific gravity and serum electrolytes. In patients with corticotroph deficiency, the oral replacement therapy was perioperatively increased in a standard manner. In patients with intact pituitary function, hormone levels were controlled before hospital discharge.

The follow-up assessments included imaging controls as well as ophthalmological and endocrinological examinations after 3 months and every other year thereafter. Ophthalmological and endocrinological assessments were performed either by the appropriate departments in our medical centre or by specialised local physicians. The diagnostic findings were checked within annual presentations in our outpatients’ department.

## Results

### Patient characteristics and previous treatments

Twelve patients with histologically confirmed craniopharyngioma WHO grade I were treated with CVC in our department between 04/2013 and 05/2017 (seven male, five female). In the case of previously missing histological confirmation of craniopharyngioma, a biopsy was taken during CVC implantation. The median age at initial tumour diagnosis was 66.5 years (range 6–75 years), with a median age at treatment with CVC of 69.0 years (range 35–76 years). Detailed patient characteristics are listed in Table 1.

**Table 1:** Patient characteristics and volumetric and ophthalmological results (*Histological confirmation pre or within CVC*, histological confirmation prior to or within CVC implantation; *FU*, at follow-up assessment; *PFS*, progression-free survival; *RE*, right eye; *LE*, left eye; *BE*, both eyes)

pat. no.	Age (years)	Sex (m/f)	Histological confirmation		Radiotherapy		Volumetric analysis (cm^3^)					PFS (months)	Ophthalmological outcome					
							Cyst volume			Tumour volume			Visual acuity				Visual field defects	
			pre CVC	within CVC	pre CVC	post CVC	preop.	postop.	FU	preop.	FU		RE preop.	RE postop.	LE preop.	LE postop.	preop.	postop.
1	75	m		x		x	5.00	0.67	0.49	9.51	1.07	22	0.8	1.0	0.7	1.0	Central scotoma (BE)	Stable (BE)
2	67	f		x		x	16.85	1.27	0.64	41.30	0.73	64	0.6	0.6	0.7	0.8	Incomplete bitemporal hemianopia (BE)	Improved (BE)
3	74	f	x			x	3.09	0.00	0.00	5.53	0.88	55	0.6	1.0	1.0	1.0	Complete left homonymous hemianopia (BE)	Markedly improved on RE, improved on LE
4	35	m	x		x		3.59	2.22	10.75	7.67	25.32	18	0.05*	0.05*	0.6	0.6	Complete right homonymous hemianopia (BE) + complete superior and incomplete inferior nasal quadrantanopia (RE)	Stable (BE)
5	45	f	x			x	28.07	20.92	3.96	29.21	4.02	58	0.0*	0.0*	0.7	--***	Complete left hemianopia (LE)	--***
6	71	m		x		x	0.59	0.07	0.00	2.05	0.10	41	0.8	0.8	0.4	0.5	Incomplete bitemporal hemianopia (BE, LE>RE)	Markedly improved (BE)
7	76	m	x			x	1.54	0.22	0.10	5.04	0.74	50	0.4	1.0	0.3	0.8	Complete bitemporal superior quadrantanopia (BE)	Markedly improved (BE)
8	74	m		x		x	6.32	1.45	0.94	8.87	6.68	9	0.2	0.3	0.5	0.7	Complete left homonymous hemianopia (BE)	Stable on RE, improved on LE
9	57	m	x		x		10.02	0.22	0.20	10.53	0.51	41	0.05**	0.05**	1.0	1.0	No defect	No defect
10	62	f	x			x	3.12	0.52	0.41	9.50	0.96	16	0.5	0.8	0.5	0.8	Incomplete bitemporal hemianopia (BE)	Improved on RE, markedly improved on LE
11	74	f		x	x		6.44	2.77	0.09	10.20	2.77	36	0.8	0.8	0.5	0.7	Incomplete bitemporal hemianopia (BE, LE>RE)	Markedly improved (BE)
12	35	m	x		x		8.16	3.84	0.29	15.90	2.58	42	1.0	1.0	1.0	1.0	No defect	No defect

*Pre-existing optic atrophy with (nearly) amaurosis on the affected eye, **pre-existing nearly amaurosis due to prior retinal detachment, ***lack of postoperative ophthalmological data

Due to the natural heterogeneous course of the disease, our patient population had several prior surgical interventions: one patient had an endoscopic cyst fenestration, two patients had been treated via an open surgical approach, and a previous stereotactic cyst puncture or implantation of an Ommaya system for subcutaneous cyst aspiration had been performed in nine patients prior to CVC.

### Surgery and postoperative course

The mean duration of surgery was 46.8 min (± 10.6 min). The mean postoperative hospital stay was 4.3 days (± 1.4 days). The intraoperative course of surgeries was uneventful, with no surgery-related complications except one small asymptomatic intracerebral haemorrhage along the surgical trajectory without mass effect or treatment demand (patient no. 9). Two patients underwent stereotactic operative revision to correct the catheter position when follow-up MR imaging showed progression of the initially reduced cyst volume with dislocation of catheter tips within the cyst walls (patient nos. 1 and 2).

Three patients had been treated with external beam radiotherapy (patient nos. 4, 9 and 11) and one patient (patient no. 12) with iodine-125 brachytherapy before CVC implantation, and the others received adjuvant radiotherapy within 6 months after CVC implantation. Intensity-modulated fractionated stereotactic radiotherapy was performed in all patients (except patient no. 12) with a total radiation dose of 52.5–54.0 gray (Gy) in 30 fractions (dose per fraction: 1.75–1.8 Gy).

### Outcome

The median clinical and radiological follow-up period was 38.5 months (± 15.3 months) and 41.0 months (± 17.1 months), respectively. Volumetric data of tumour and cyst volumes, as well as ophthalmological and endocrine parameters, were retrospectively analysed before and after surgery, and at latest follow-up.

#### Volumetric outcome

**Fig. 4:**
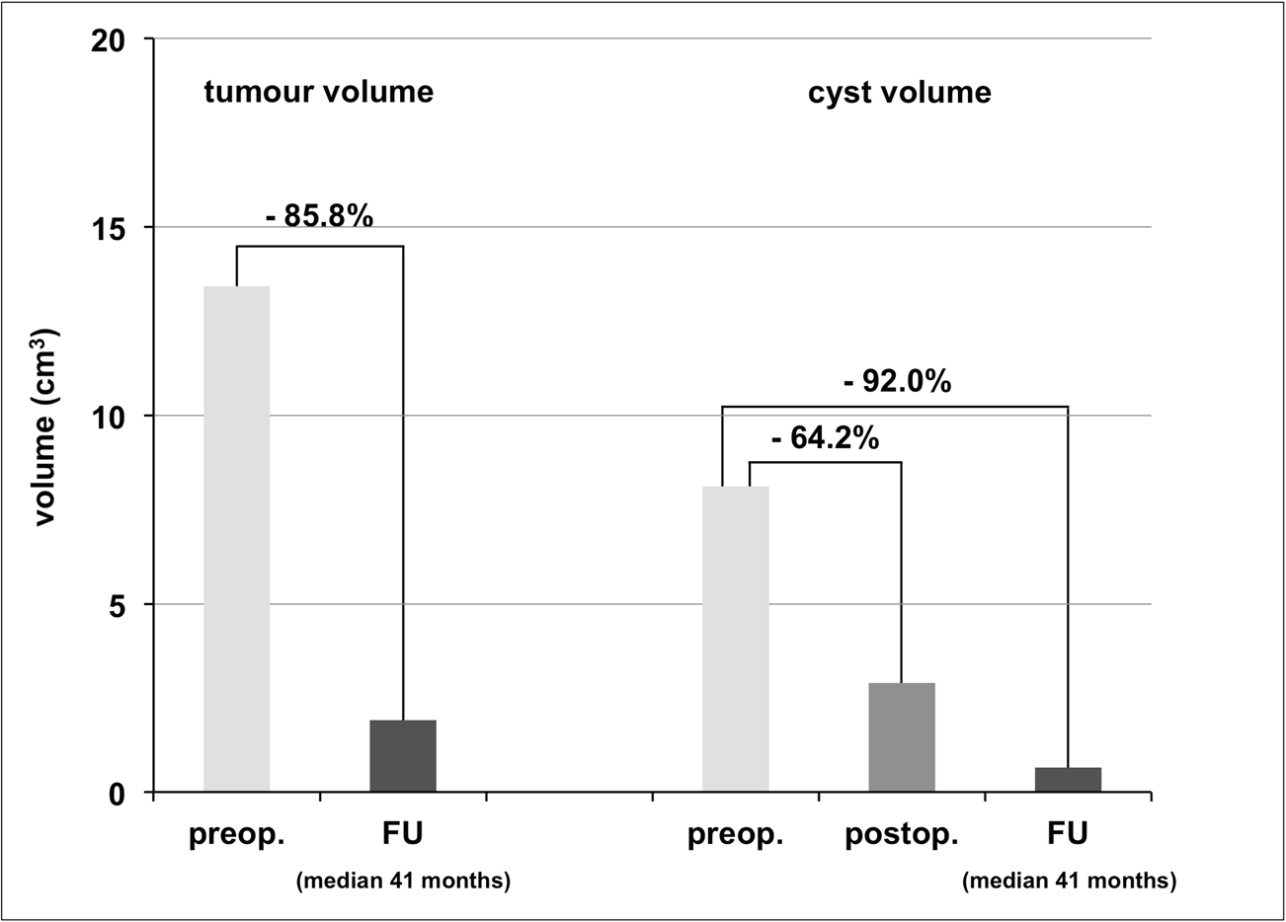
Volumetric analysis (preoperatively = light grey, postoperatively = medium grey, at follow-up assessment (FU) = dark grey, mean values of 11/12 patients excluding patient no. 4). Mean reduction of the total tumour volume of 85.8% at follow-up assessments, and mean reduction of cyst volume of 64.2% after surgery and 92.0% at follow-up assessments, each compared to the corresponding preoperative values

Volumetric analyses demonstrated a mean postoperative reduction of cyst volume of 64.2% compared to preoperative values. At follow-up assessments after completed radiotherapy, there was a mean reduction of cyst volume of 92.0% and total tumour volume (including solid tumour and cysts) of 85.8% compared to preoperative values, as shown graphically in Fig. 4 (patient no. 4 excluded, see below). The individual volumetric values, as well as the progression-free survival periods of each patient, are listed in Table 1. No patient in our group developed cyst regrowth or tumour progression during the follow-up period except patient no. 4. The median progression-free survival period of all patients was 41.0 months (± 17.1 months).

Patient no. 4 had been treated radiotherapeutically before CVC implantation and showed massive tumour progression with multiple new cysts in the last follow-up MRI 18 months after surgery. Due to the large solid and cystic tumour volume and consecutive displacement of CVC, the initially drained cyst was hard to distinguish; therefore, volumetric measurements of patient no. 4 are listed in Table 1 but were excluded from the summarised data shown in Fig. 4.

#### Ophthalmological outcome

Preoperative tumour-associated visual reduction was present in ten patients, nine of which showed improvement after surgery; patient no. 5 missed the postoperative examination, but she reported subjective improvement. Two patients without preoperative tumour-associated visual reduction remained stable without deterioration. The mean visual acuity of the right and left eyes improved after surgery from 0.48 to 0.62 and from 0.65 to 0.81, respectively.

Preoperative tumour-associated visual field defects improved in seven out of ten affected patients after surgery (two patients with full and five patients with partial recovery). Two affected patients and two non-affected patient remained stable without deterioration. No postoperative assessment of visual fields was available for patient no. 5.

The ophthalmological values of all patients are listed in Table 1.

#### Endocrinological outcome

Endocrine disorders remained stable without deterioration. Pre-existing anterior pituitary gland deficiency was present in eight patients (corticotroph axis: five patients, thyreotroph axis: seven patients, gonadotroph axis: four patients, somatotroph axis: one patient). Pre-existing posterior pituitary gland deficiency or hypothalamic disorder was present in two or three patients, respectively.

## Discussion

For decades, the first-line therapy of craniopharyngiomas was radical surgery and gross total resection [36]. Due to the tumour invasiveness and proximity or adherence to critical neurovascular structures, H. Cushing described complete surgical removal as one of the most baffling problems faced by neurosurgeons with a high risk to produce severe secondary symptoms [8, 30]. Despite the present advancement of modern microsurgery, complete tumour removal even by expert hands still carries a relevant risk of surgery-associated damage to the visual system, as well as hypothalamic-pituitary structures with consecutive symptoms significantly lowering the quality of life [10, 22, 43]. Different strategies have been developed to address the issue of maximal local tumour control and best preserving functional integrity [29]. Subtotal resection combined with radiotherapy was established as a reasonable concept to achieve tumour control rates similar to those for gross total resection but with reduced treatment-related morbidity, hence, improved quality of life [10, 43].

Due to the heterogeneous and chronic nature of craniopharyngioma, there were several previous treatments in our group before CVC implantation, like transcranial resection, endoscopic fenestration, stereotactic puncture or implantation of Ommaya systems and prior radiotherapy. In selected cases of primarily solid craniopharyngiomas, less so in cystic tumours, fractionated stereotactic radiotherapy alone offers promising results regarding local tumour control and functional preservation [7, 13]. Even if the solely radiotherapy-induced side effects on the visual or endocrinological system are difficult to quantify, the visual system seems to be at low risk, given the modern radiotherapeutic methods [13]. Like with any surgical intervention, endocrinological dysfunction is not about to improve, and additional endocrinopathies as well as deterioration of hypothalamic dysfunction might occur [5, 13, 41].

The vast majority of craniopharyngiomas contain cystic formations frequently leading to the compression of the surrounding neurovascular structures [6, 19, 27, 31]. Additionally, several studies have reported cyst enlargement due to radiotherapy in as many as 30–60% of craniopharyngioma patients [1, 16, 38]. Accompanying neurological deterioration requiring surgical interventions for cyst volume reduction is not uncommon [29]. Several endoscopic approaches have been described with their respective limitations, mainly due to the cyst location and the problem of cyst recurrence [12, 18, 42]. With the trend towards more conservative surgical strategies, stereotactic approaches have become increasingly important [2, 4, 10, 25, 33]. In the 1960s, Leksell et al. first developed a minimally invasive treatment concept combining stereotactic methods with ionising radiation to manage craniopharyngiomas [20]. In consideration of the high grade of accuracy and instrument stability, stereotactic approaches were later extended for other treatment options, like cyst punctures, implantation of Ommaya systems or intracystic drug injections, as well as to delay definitive surgery or radiotherapy in children for several years via consecutive cyst shrinkage [3, 40, 45]. To address the problem of cyst recurrence, intracystic catheter placement with subcutaneous reservoirs allowed repeated cyst aspirations [1, 39]. Besides causing neurological problems, fluctuating cyst sizes could also hinder radiotherapeutic treatment planning, here, stable and smallest possible conditions are preferable.

Several authors described the rather incidental finding of the long-term reduction of cyst volume by fenestration to cerebrospinal fluid (CSF) spaces [9, 17, 21, 34]. Following Laplace’s law of fluid mechanics, the fluid content of craniopharyngioma cysts, with a considerably smaller total volume compared to the cisternal or ventricular CSF system, seems to be spontaneously and permanently drained via appropriate connections. In 1989, Spaziante et al. described the phenomenon of progressive spontaneous reduction of cyst size after connecting cyst cavities to ventricular and/or cisternal CSF spaces in their series of six craniopharyngioma patients [39]. In 2013, Moussa et al. published data regarding 52 patients with mainly monocystic craniopharyngioma treated with Ommaya reservoir catheters with initial cyst aspiration. Seventy-three percent of their patients did not develop any recollection of cysts over a follow-up period of 7 years. The authors hypothesised a constant egress of cyst fluid via the terminal holes of the catheters to the subarachnoid space after the initial collapse of the cysts [21].

Following these observations, we investigated the specific effect of stereotactic transventricular implantation of CVC intending communication between the cyst cavity and ventricular system to reduce and control cystic components in craniopharyngioma patients. The minimally invasive procedure offered the advantage of a short mean duration of surgery with 46.8 min as well as a short mean postoperative hospital stay of 4.3 days. The intraoperative course was uneventful, and besides one small asymptomatic bleeding along the surgical trajectory, no further surgery-related problems occurred. Moreover, there was no additional treatment-related morbidity concerning the visual system or the endocrinological or hypothalamic function. Other authors reported similar results regarding their minimally invasive treatment methods [9, 21, 36, 39].

Problems with penetration of a possibly rough cyst capsule or secondary dislocation of the inserted catheter tip due to cyst shrinkage after initial aspiration are described for stereotactic procedures with the potential need for revision surgery [39, 46]. In our series, we also observed secondary dislocation of the catheter tip at the margin of the cyst wall after intraoperative cyst aspiration in the first two patients treated with CVC. The consecutive cyst refilling showing up on follow-up MR images led to revision surgery after several weeks. Further on, the position of the catheter tip within the cyst cavity was planned as deep as possible on the stereotactic trajectory to prevent this problem, thereby maintaining the position of the catheter even when the whole cyst content was aspirated. After this adaption, there were no further cases of catheter dislocation or cyst refilling with a correctly positioned catheter during our median follow-up period of 41 months. Catheter obstruction with debris due to the high protein content of the cyst fluid seems another possible complication, though this problem did not occur in the presented series. In these cases, further open or endoscopic surgical interventions may be required to achieve sufficient cyst fenestration.

No major complications occurred during our follow-up period; in particular, there were no cases of chemical meningitis or consecutive hydrocephalus (due to possible alterations of CSF flow or resorption rates) despite draining of cyst fluid into the CSF space. This is in line with other reports and might be attributed to the high dilution of cyst fluid in the whole CSF space [21, 36, 39]. Also, no spreading of tumour cells throughout the subarachnoid space was observed in previous publications [21, 36, 39] or in our series, which may be explained by the usual lack of tumour cells in the cyst fluid and the benign nature of the tumour.

The described method was highly efficient in the permanent reduction of the drained cysts. Volumetric analyses demonstrated a mean postoperative reduction of cyst volume of 64.2% compared to preoperative values. In our selected patient cohort, this proved to be a stable long-term effect with a mean cyst volume reduction of 92.0% and a mean total tumour volume (including solid tumour and cysts) reduction of 85.8% at last follow-up assessments compared to preoperative values. The postoperative reduction of cyst size provided stable conditions for adjuvant radiotherapy. The long-term reduction of cyst size might be a combined effect of cysto-ventriculostomy and the antineoplastic radiotherapeutic effect.

Similar results with high rates of progression-free survical concerning cyst sizes are described in several case series mentioned above, in particular, in context of different strategies connecting cyst cavities to the subarachnoid space [21, 28, 32]. Rachinger et al. performed a volumetric assessment of preoperative tumour volumes, but did not report on the development of volumes in the postoperative course [34]. To our knowledge, we are the first to describe the development of cyst and tumour volumes of cystic craniopharyngiomas under stereotactic treatment with CVC. This differentiated assessment is therefore possible because in our specific patient cohort, only patients without further surgical treatments within the follow-up period were included. This allows us to make a selective statement about the safety and efficacy of the described stereotactic method.

About 75% of patients diagnosed with craniopharyngioma suffer from associated ophthalmological symptoms. In our patient group, visual impairment was improved in most patients following cyst reduction via CVC implantation in the immediate postoperative course, with no patients showing deterioration of visual status. Hence, the authors estimate high recovery rates up to 90% following tumour resection strategies and also surgery-related visual deterioration in about 15% of patients [12, 35]. Minimally invasive approaches for cyst reduction seem superior in achieving immediate visual improvement given the high rate of recovery and the near absence of new ophthalmological deficits [28, 36].

Consistent with other reports regarding minimally invasive therapeutic strategies [21, 28, 36], there was neither improvement nor deterioration of endocrinological dysfunction in our patients after CVC implantation. One of our patients suffered from a new onset of complete pituitary dysfunction 18 months after CVC implantation and adjuvant radiation therapy without new imaging aspects; therefore, this development could not be attributed to surgery. In contrast, tumour resection via the transcranial or transsphenoidal approach carries a high risk for endocrinological worsening with surgery-related new complete or incomplete pituitary dysfunction in over 50% of cases compared to minimally invasive strategies [15, 35, 37].

The ideal management of craniopharyngiomas is still controversial and challenging. Nowadays, there is a tendency away from aggressive high-risk strategies towards less invasive methods aimed at symptom relief, long-term tumour control and maintaining quality of life. The varying presentation and behaviour of these relatively rare tumours affecting a heterogeneous group of patients make it difficult to perform prospective randomised trials; thus, the publication of different therapeutic trials, even in small patient groups, seems especially important in this context giving new perspectives on patient management and the discussion of individualised treatment concepts.

Stereotactic CVC implantation proved to be a safe minimally invasive technique for the treatment of selected patients with cystic craniopharyngiomas. The risk of procedure-related complications was low and no case of deterioration of pituitary dysfunction or additional surgery-related hypophyseal or hypothalamic disorders was observed. The method was highly effective in cyst size reduction and consecutive ophthalmological improvement. Additionally, the permanent reduction of cyst size enabled enhanced and stable conditions to perform radiotherapy to further provide long-term local tumour control. Stereotactic CVC implantation seems promising in achieving acute symptom relief and long-term local tumour control with preservation or even improvement of quality of life. The small number of patients in our selected group is certainly a limitation of our study. However, this is a common problem in literature dealing with craniopharyngiomas, in particular, with the issue of minimally invasive concepts due to the rareness and heterogeneous nature of this disease.

## Conclusions

Stereotactic CVC implantation proved to be a safe minimally invasive technique for the treatment of craniopharygioma cysts leading to a long-term reduction of cyst volumes with optimised conditions for radiotherapy and an improvement of visual symptoms. Combined with adjuvant radiotherapy, we consider this method as suitable for first-line therapy of cystic craniopharyngiomas, particularly in elderly patients with comorbidities impeding more invasive strategies.

## Data Availability

The anonymized datasets analyzed during the current study are available from the corresponding author on reasonable request.
